# Effectiveness of teleassistance on the improvement of health related quality of life in people with neuromuscular diseases

**Published:** 2012-06-15

**Authors:** Oscar Martínez, Amaia Jometón, Esther Lázaro, Imanol Amayra, Juan Francisco López-Paz, Manuel Pérez, Patricia Caballero, Luís De Nicolás, Alberto Lasa, Jorge Roldán, Natalia Martín, Joseba Bárcena, Luís Varona

**Affiliations:** Neuromuscular and Neurodevelopment Disorders Research Group (Neuro-e-Motion), Faculty of Psychology and Education, Department of Personality Assessment and Psychological Interventions, University of Deusto, Bilbao, Spain; Neuromuscular and Neurodevelopment Disorders Research Group (Neuro-e-Motion), Faculty of Psychology and Education, Department of Personality Assessment and Psychological Interventions, University of Deusto, Bilbao, Spain; Neuromuscular and Neurodevelopment Disorders Research Group (Neuro-e-Motion), Faculty of Psychology and Education, Department of Personality Assessment and Psychological Interventions, University of Deusto, Bilbao, Spain; Neuromuscular and Neurodevelopment Disorders Research Group (Neuro-e-Motion), Faculty of Psychology and Education, Department of Personality Assessment and Psychological Interventions, University of Deusto, Bilbao, Spain; Neuromuscular and Neurodevelopment Disorders Research Group (Neuro-e-Motion), Faculty of Psychology and Education, Department of Personality Assessment and Psychological Interventions, University of Deusto, Bilbao, Spain; Neuromuscular and Neurodevelopment Disorders Research Group (Neuro-e-Motion), Faculty of Psychology and Education, Department of Personality Assessment and Psychological Interventions, University of Deusto, Bilbao, Spain; Neuromuscular and Neurodevelopment Disorders Research Group (Neuro-e-Motion), Faculty of Psychology and Education, Department of Personality Assessment and Psychological Interventions, University of Deusto, Bilbao, Spain; Neuromuscular and Neurodevelopment Disorders Research Group (Neuro-e-Motion), Faculty of Psychology and Education, Department of Personality Assessment and Psychological Interventions, University of Deusto, Bilbao, Spain; Neuromuscular and Neurodevelopment Disorders Research Group (Neuro-e-Motion), Faculty of Psychology and Education, Department of Personality Assessment and Psychological Interventions, University of Deusto, Bilbao, Spain; Neuromuscular and Neurodevelopment Disorders Research Group (Neuro-e-Motion), Faculty of Psychology and Education, Department of Personality Assessment and Psychological Interventions, University of Deusto, Bilbao, Spain; University of Valladolid, Faculty of Education and Social Work, Department of Psychology, Valladolid, Spain; Hospital of Cruces, Barakaldo, Spain; Hospital of Basurto, Bilbao, Spain

**Keywords:** teleassistance, online support, neuromuscular disease, isolation, quality of life

## Abstract

**Background:**

Neuromuscular diseases are a group of pathologies characterized by the progressive loss of muscular strength, atrophy or hypertrophy, fatigue, muscle pain and degeneration of the muscles and the nerves controlling them (The French Muscular Dystrophy Association, 2004). Perceived isolation and health related quality of life are affected in the majority of cases due to the illness chronicity. Internet, and in this way, the use of chat and videoconferencing programs, is an alternative option to mitigate the mentioned variables.

**Aim:**

The aim of the study is to assess the effectiveness of teleassistance on reducing isolation and improving health related quality of life in adults with neuromuscular diseases.

**Methods:**

The sample is composed of 60 participants randomly selected and affected by different neuromuscular diseases (e.g. Myasthenia Gravis, Becker Muscular Dystrophy, Facioescapulohumeral Muscular Dystrophy, etc.). Thirty patients were assigned to the experimental group, which participated in the chat and videoconferencing sessions, and the other thirty to the control group, which did not participate. The inclusion criteria for both groups were: medical confirmed diagnosis (CIE-10) of one of the diseases mentioned above, age ≥18 years, agreeing to participate in the study by signing an informed consent, and finally, ability to manipulate a computer (just for the experimental group). The exclusion criteria for both groups were to have a psychiatric disorder (DSM-IV-TR), head trauma or severe visual limitations. All the patients were recruited from neuromuscular disorders associations and Hospitals of The Basque Country. Effectiveness were assessed by a pre-post design in which questionnaires and interviews were administrated (e.g. Disability Assessment Schedule—WHO-DAS II, Sickness Impact Profile, The MOS Social Support Survey, etc.). The online support entails different activities developed during three months in once a week sessions: a) Group videoconference sessions with a Psychologist, b) Individual videoconference sessions with a Neurologist, and c) Forum discussion groups about biopsychosocial issues. The psychologist counseling consists on a psychosocial program about general topics such as illness information, emotional reactions to the disease, the most frequently automatic thoughts, etc. A web site was developed to carry out the intervention: http://neuromusculares.deusto.es/. An exhaustive preliminary analysis of this pre-post assessment is necessary in order to know if the psychosocial programme is effective and if it could be a helpful tool for this type of population.

**Results:**

Preliminary results will be presented in order to confirm if teleassistance is an effective alternative way of advising people with neuromuscular disorders.

